# European Bilberry Extract Ameliorates Dietary Advanced Glycation End Products-Induced Non-Alcoholic Steatohepatitis in Rats via Gut Microbiota and Its Metabolites

**DOI:** 10.3390/nu17243918

**Published:** 2025-12-15

**Authors:** Lihui Shen, Ruijie Cheng, Wenwen Chen, Hongjie Liu, Xinyu Wang, Ruikun He, Xiaoxing Mo, Liegang Liu

**Affiliations:** 1 Department of Nutrition and Food Hygiene, Hubei Key Laboratory of Food Nutrition and Safety, School of Public Health, Tongji Medical College, Huazhong University of Science and Technology, 13 Hangkong Road, Wuhan 430030, China; d202482056@hust.edu.cn (L.S.); d202181656@hust.edu.cn (R.C.); m202375749@hust.edu.cn (W.C.); d201981405@hust.edu.cn (H.L.); xywang_@hust.edu.cn (X.W.); 2 Ministry of Education Key Lab of Environment and Health, School of Public Health, Tongji Medical College, Huazhong University of Science and Technology, 13 Hangkong Road, Wuhan 430030, China; 3 BYHEALTH Institute of Nutrition & Health, Guangzhou 510663, China; herk@by-health.com

**Keywords:** European bilberry extract, advanced glycation end products, microbiota, short-chain fatty acid, GPR43

## Abstract

**Background:** Gut dysbiosis is implicated in the pathogenesis of non-alcoholic steatohepatitis (NASH) caused by diets rich in advanced glycation end products (AGEs). European bilberry extract (EBE) exerts a regulatory effect on gut microbiota. Nevertheless, it is still unknown whether EBE influences NASH via gut microbiota and their metabolites. This study aimed to investigate the effects and underlying mechanisms of EBE on NASH caused by a long-term AGEs diet. **Methods:** Rats fed with a high-AGE diet were orally administered with EBE for 80 weeks, and NASH was measured. 16S rRNA analysis and targeted metabolomics were used to detect gut microbiota and SCFA, respectively. The hepatic expression of SCFA receptors and that of the HMGB1/RAGE/NF-κB signaling pathway were detected to investigate the possible molecular mechanism. **Results:** EBE reduced the accumulation of AGEs in the circulation and liver of high-AGE diet-fed rats. EBE also ameliorated impaired glucose tolerance and insulin sensitivity, liver inflammation, steatosis, fibrosis, and dysfunction in high-AGE-fed rats. EBE reshaped high-AGE diet-induced gut dysbiosis by increasing short-chain fatty acid (SCFA)-producing bacteria and SCFA levels and reducing deleterious bacteria. Mechanistically, EBE promoted the activation of GPR43 and inhibited the activation of downstream HDAC3 and HMGB1/RAGE/NF-κB signaling pathway in the liver of high-AGE diet-fed rats. Additionally, EBE decreased the levels of TNF-α, IL-1β, and IL-6 and increased the level of IL-10 in the liver of high-AGE diet-fed rats. **Conclusions:** EBE promoted the production of SCFA, which might engage with the GPR43 receptor and inhibited the activation of HDAC3 and HMGB1/RAGE/NF-κB signaling pathway, ultimately alleviating NASH caused by a high-AGE diet.

## 1. Introduction

Nonalcoholic fatty liver disease (NAFLD) is the most prevalent liver disease worldwide. NAFLD encompasses extensive liver damage, ranging from steatosis to nonalcoholic steatohepatitis (NASH) [1]. NASH is mainly characterized by lipid accumulation in hepatocytes and inflammatory cell infiltration, which may further develop into liver cirrhosis, hepatocellular carcinoma, and liver failure [2]. Diets are important environmental factors affecting the development of NASH [3]. Advanced glycation end products (AGEs) are compounds formed during the Maillard reaction between reducing sugars and the free amino groups of proteins, lipids, or nucleic acids in food [4]. The accumulation of AGEs in the body is implicated in the onset and progression of various chronic disorders [5]. Exogenous advanced glycation end products (AGEs), particularly heat-treated foods, constitute the primary contributor to the human AGE reservoir [6]. Through their interaction with RAGE, AGEs trigger downstream signaling cascades like NF-κB, thereby promoting the secretion of inflammatory mediators and ultimately inducing liver injury [7]. Nonetheless, existing evidence focuses on the short-term effect of dietary AGEs on liver damage [8,9]; the role of long-term dietary AGEs in liver health remains unclear.

Gut microbiota and its metabolites are involved in the process of dietary AGEs-induced chronic diseases [10]. After ingestion, a minority (10–30%) of AGEs are absorbed, while the rest are metabolized by gut microbiota [11]. Dietary AGE-induced pathological damage in the liver is accompanied by the dysbiosis of the gut microbiota and its metabolites [12,13]. In contrast, probiotics treatment alleviates nonalcoholic fatty liver disease in AGEs-treated mice [14]. Short-chain fatty acids (SCFAs), a class of characteristic microbial metabolites originating from carbohydrates, have antagonistic effects on chronic diseases [15]. SCFAs engage with their receptors, including GPR43 and GPR109a, to suppress the NF-κB signaling cascades, thereby further reducing pro-inflammatory reactions and tissue injury [16]. Thus, targeting the gut microbiota and its metabolites might be a promising approach to counteract chronic disorders caused by dietary AGEs.

Plant extracts, such as organic acids, plant essential oils, and anthocyanins, are now considered a promising strategy for preventing NASH [17]. European bilberry extract (EBE), rich in anthocyanins, exhibits diverse biological activities such as anti-inflammatory, antioxidant, and immune enhancement [18]. Due to the poor bioavailability of anthocyanins, their absorption and metabolism are mainly affected by the gut microbiota [18]. Evidence indicates that the health benefits of anthocyanins are closely linked to the gut microbiota and its related metabolites [19]. Moreover, blueberry and blackberry anthocyanins ameliorate high-fat diet-induced NAFLD through modulating the gut microbiota and promoting the production of SCFA [20]. Furthermore, our previous study has demonstrated that EBE could ameliorate AD-like pathological changes induced by a high-AGE diet [21]. Nevertheless, it is unclear whether EBE exerts its role in dietary AGEs-induced liver damage through the gut microbiota and SCFA.

In this study, a rat model of long-term high-AGE feeding was established to investigate its effects on liver health. The potential effect and underlying mechanism of EBE on long-term dietary AGEs-induced liver damage were also investigated from the perspective of SCFA and their receptors.

## 2. Materials and Methods

### 2.1. Materials

The EBE (ACNN15^®^, supplied by By-Health Co., Ltd. Guangzhou, China) comprised a mixture of fifteen anthocyanins and five anthocyanidins. The complete chemical profile of this compound was reported previously [22].

### 2.2. Animals

Thirty female Sprague–Dawley rats (12 weeks old) were supplied by Vital River Laboratory Animal Center (Beijing, China). The animals were housed under specific pathogen-free conditions with a controlled temperature and a 12 h:12 h light-dark cycle. Food and water were available ad libitum.

The study was approved by the Institutional Animal Care and Use Committee at Tongji Medical College, Huazhong University of Science and Technology, and performed in accordance with the National Research Council’s Guide for the Care and Use of Laboratory Animals (Permission ID:2519, date on 15 July 2020).

### 2.3. Preparation of High-AGE Diet

The high-AGE diet was produced by baking standard chow (AIN-93M, Medison Biopharmaceutical Co., Ltd. Yangzhou, China) at 150 °C for 30 min, following an established protocol [22]. The nutritional and caloric composition of the standard and high-AGE diets were available in our previous study [22].

### 2.4. EBE Intervention

After a one-month acclimation period, rats were randomly allocated into three groups (*n* = 10 each), namely control, high-AGE diet, and EBE groups. The EBE group received the high-AGE diet and a daily oral gavage of 150 mg/kg EBE. The AGE group received the high-AGE diet and a daily oral gavage of vehicle (normal saline). The control group received the standard chow diet and a daily oral gavage of vehicle. Following 80 weeks of intervention, feces were collected for 16S rRNA analysis and SCFA detection. Rats then underwent oral glucose tolerance test (OGTT) and intraperitoneal insulin tolerance test (iPITT). After an overnight fast, the rats were anesthetized with isoflurane (5% *v*/*v*) and sacrificed via descending aorta blood collection. Serum was obtained to measure SCFA, AGE, and liver function biomarkers. Sections of liver tissue were either fixed in 4% paraformaldehyde for pathological examination or snap-frozen for subsequent downstream analysis. Throughout the study, body weight was measured twice weekly, and food intake was recorded daily. Four rats in the control group, five in the high-AGE diet group, and five in the EBE group were excluded due to mortality from causes such as tumors, intestinal obstruction, or chronic infection, as assessed by veterinarians.

### 2.5. OGTT

Following an overnight fast, blood glucose levels were measured at baseline. The rats were then orally gavaged with a 2 g/kg dose of glucose (20% solution; Sigma-Aldrich, St. Louis, MO, USA). Blood glucose levels were subsequently monitored at 15, 30, 45, 60, 90, and 120 min post-administration. Glucose tolerance was assessed by calculating the area under the curve (AUC) value.

### 2.6. iPITT

Following a 6 h fast, blood glucose levels were measured at baseline. Then, rats were intraperitoneal injected with 0.75 U/kg insulin (Novo Nordisk, Copenhagen, Denmark). Blood glucose levels were subsequently detected at 15, 30, 45, 60, 90, and 120 min post-administration. Insulin sensitivity was assessed by calculating the AUC value.

### 2.7. AGEs Measurements

The concentrations of two major AGEs, *N*^ε^-carboxymethyllysine (CML) and *N*^ε^-carboxyethyllysine (CEL), in diet, serum, and liver samples were quantified using ultra-high performance liquid chromatography-tandem mass spectrometry (UHPLC-MS/MS) according to our established methodology [22]. These AGEs were analyzed in both free and bound forms.

For the free forms, serum was mixed with the internal standard and deproteinized using methanol and acetonitrile. After centrifugation, the supernatant was derivatized with Nonafluorovaleric Acid aqueous solution for UHPLC-MS/MS analysis. For the bound forms, serum was reduced with sodium borohydride and precipitated with trichloroacetic acid. After centrifugation, the collected precipitates were combined with the internal standard and hydrolyzed in hydrochloric acid. The hydrolysate was then concentrated under a nitrogen stream, reconstituted in pure water, and centrifuged. The resulting supernatant was finally derivatized with Nonafluorovaleric Acid aqueous solution prior to UHPLC-MS/MS analysis. Diet and liver samples were homogenized in water and processed following the same procedure as serum.

### 2.8. Measurements of Serum Liver Function Parameters and Liver Inflammatory Cytokines

Hepatic total cholesterol (TC) and triglyceride (TG) levels, along with serum alanine aminotransferase (ALT) and aspartate transaminase (AST) activities, were quantified using commercial assay kits (Elabscience, Wuhan, China) according to the manufacturers’ protocols. The levels of tumor necrosis factor (TNF)-α, interleukin (IL)-1β, IL-6, and IL-10 in the liver were measured using Elisa kits (R&D Systems, Minneapolis, MN, USA) as per the provided protocols.

### 2.9. 16S rRNA Analysis

Fecal microbial DNA was extracted using a commercial kit (TIANGEN Biotech Co. Ltd., Beijing, China) and quantified with NanoDrop™ ND-2000 (Thermo Fisher Scientific Ltd., Waltham, MA, USA). The V3-V4 hypervariable region of the 16S rRNA gene was amplified with primers V3F and V4R, and the resulting amplicons were sequenced on an Illumina MiSeq platform (Illumina, San Diego, CA, USA). Sequencing reads were processed in QIIME (v.1.9.1), where they were clustered into operational taxonomic units (OTUs) at a 97% similarity threshold for downstream analysis.

The β diversity was performed by non-metric multidimensional scaling (NMDS) analysis based on the weighted Unifrac distance algorithm. Intergroup differences in microbial composition at various taxonomic levels were evaluated based on relative abundance, with a false discovery rate (FDR) < 0.05 considered significant. The heatmap was generated by the ggplot2 R package (version 4.3.0, RStudio, Boston, MA, USA).

### 2.10. SCFA Detection

The levels of SCFA in feces and serum were measured by gas chromatography–mass spectrometry/mass spectrometry analysis (GC-MS/MS, Agilent Technologies, Santa Clara, CA, USA) as previously described [23]. Briefly, fecal samples were homogenized in ultrapure water and centrifuged to collect the supernatant. Aliquots of both the fecal supernatant and serum were separately mixed with acetone. After centrifugation, the resulting supernatant was derivatized by mixing with acetone, 100 μL 100 mM pentafluorobenzyl bromide solution, 2-ethylbutyric acid, and phosphate-buffered saline at 60 °C for 2 h, followed by the addition of hexane. The mixture was centrifuged, and the resulting supernatant was collected for subsequent GC-MS/MS analysis.

### 2.11. Pathological Examination

Histopathological evaluation was conducted on paraffin-embedded liver sections (7–8 μm). These sections were routinely stained with hematoxylin and eosin (H&E) for the assessment of general tissue architecture and morphological changes. To specifically evaluate hepatic fibrosis, consecutive paraffin sections were stained with Sirius red according to standard protocols. Meanwhile, lipid accumulation within the liver was assessed by staining fresh frozen sections (10 μm thick) with Oil red O. All stained sections from the various procedures were systematically examined and imaged under a bright-field microscope (Olympus IX71, Tokyo, Japan). Finally, the resulting images were subjected to quantitative analysis using ImageJ (version 1.54p, Bethesda, MD, USA) software to obtain objective measurements of the pathological features.

### 2.12. Immunohistochemical Staining

The paraffin-embedded liver sections (7–8 µm) were first deparaffinized, rehydrated, and subjected to antigen retrieval. The sections were then incubated with a primary antibody against α-smooth muscle actin (α-SMA), followed by a biotin-labeled secondary antibody. The antigen–antibody complexes were visualized using a DAB kit (Zs bio, Beijing, China). Stained images were captured under an Olympus IX71 microscope and quantitatively analyzed with ImageJ (version 1.54p, Bethesda, MD, USA) software.

### 2.13. Western-Blot

Protein extraction from liver tissues was performed using RIPA lysis buffer (Beyotime Biotechnology Co., Ltd. Shanghai, China) containing a protease inhibitor cocktail. Following centrifugation, the supernatant was collected for protein quantification with a BCA kit (Beyotime Biotechnology Co., Ltd. Shanghai, China). Proteins were separated via SDS-PAGE, transferred to nitrocellulose membranes, and blocked with 5% milk. The membranes were then incubated with specific primary antibodies, followed by HRP-conjugated secondary antibodies. The primary antibodies were as follows: GPR43 (Cat #19952-1-AP, 1:1000, Proteintech (Rosemont, IL, USA)), HDAC3 (Cat# ab32369, 1:1000, Abcam (Cambridge, UK)), HMGB1 (Cat# ab79823, 1:1000, Thermo Scientific (Waltham, MA, USA)), RAGE (Cat# ab216329,1:1000, Abcam (Cambridge, UK)), *p*-NF-κB (Cat# 3033, 1:1000, CST (Danvers, MA, USA)), NF-κB (Cat# 8242,1:1000, CST (Danvers, MA, USA)), and GADPH (Cat# 3670S, 1:1000, CST (Danvers, MA, USA)). Quantitative analysis was performed with the Image J (version 1.54p, Bethesda, MD, USA) software.

### 2.14. Data Analysis

Prior to statistical analysis, data were assessed for normality and homogeneity of variance. For parametric data, comparisons between two groups were performed using a two-tailed unpaired Student’s *t*-test; multi-group comparisons employed one-way ANOVA with Tukey’s post hoc test. Non-parametric data were analyzed by the Kruskal–Wallis test followed by Dunn’s post hoc analysis. R *p* < 0.05 was regarded as significant. Data were shown as mean ± SEM.

## 3. Results

### 3.1. EBE Reduces the Accumulation of AGEs in the Circulation and Liver of High-AGE Diet-Fed Rats

**Figure 1:**
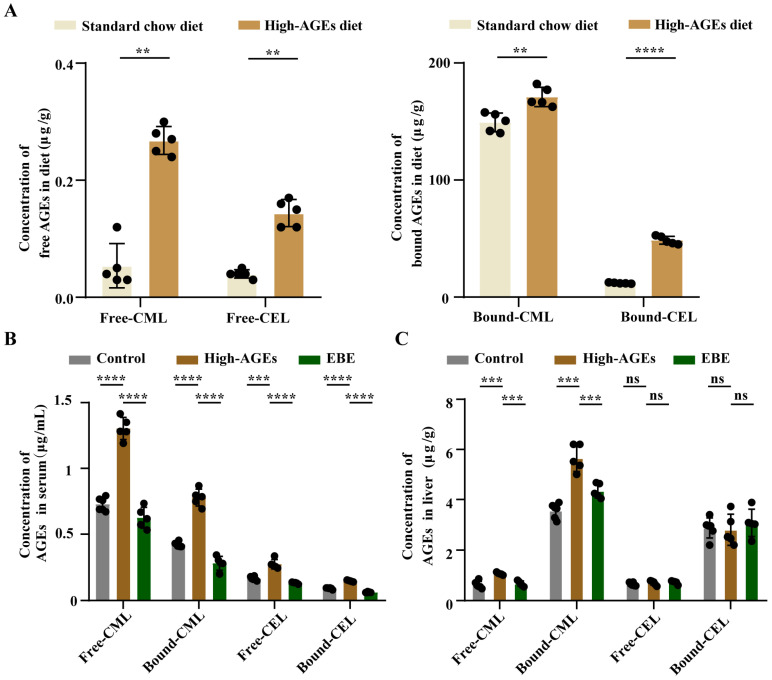
High-AGE diet leads to AGE accumulation in the circulation and liver. (**A**) The levels of AGEs in diets (*n* = 5). (**B**) The levels of AGEs in the serum (*n* = 5–6). (**C**) The levels of AGEs in the liver (*n* = 5–6). Data were expressed as mean ± SEM. Data were analyzed by the two-tailed unpaired Student’s *t*-test (**A**) or one-way ANOVA, followed by Tukey’s multiple comparisons test (**B**,**C**). ** *p* < 0.01, *** *p* < 0.001, and **** *p* < 0.0001 vs. high-AGE diet-fed rats. ns: not significant.

As shown in Figure 1A, high temperature baking significantly increased the levels of free-/bound-CML and free-/bound-CEL in diets (*p* < 0.01, Figure 1A). Consuming this high-AGE diet led to a marked elevation of free-/bound-CML and free-/bound-CEL in the circulation, and of free-/bound-CEL in the liver (*p* < 0.001, Figure 1A,B). Notably, EBE intervention attenuated the elevated levels of circulating free-/bound-CML and free-/bound-CEL caused by the high-AGE diet. (*p* < 0.001, Figure 1B). Similarly, EBE intervention also alleviated the elevated levels of free-/bound-CML in the liver caused by the high-AGE diet (*p* < 0.001, Figure 1C). There were no significant differences in the levels of free-/bound-CEL in the liver among the three groups (Figure 1C). Overall, EBE mitigated the accumulation of AGEs in the circulation and liver caused by a long-term high-AGE diet.

### 3.2. EBE Alleviates Glucose Metabolism Disorders in High-AGE Diet-Fed Rats

**Figure 2:**
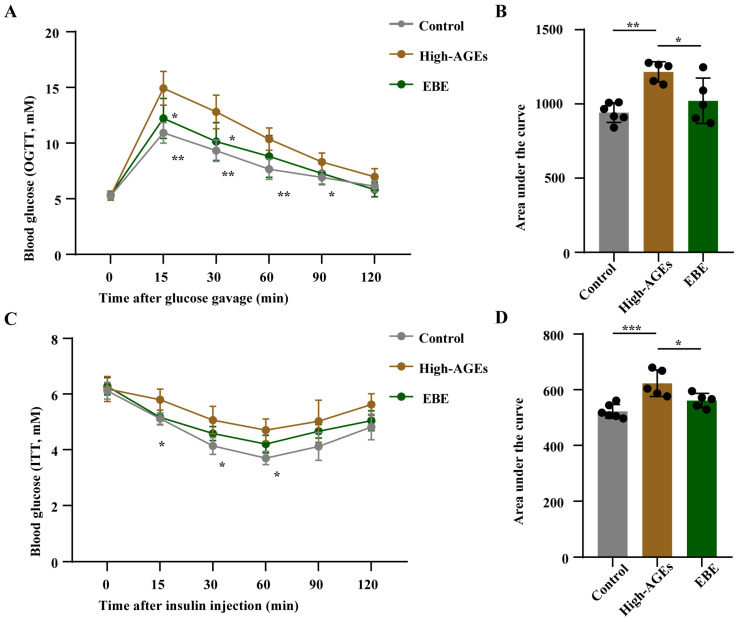
EBE intervention alleviates the impairment of glucose intolerance and insulin sensitivity in rats fed with a high-AGE diet. (**A**) The levels of blood glucose in the oral glucose tolerance test (OGTT) (*n* = 5–6). (**B**) The area under the curve (AUC) value of blood glucose in OGTT (*n* = 5–6). (**C**) The levels of blood glucose in the intraperitoneal insulin tolerance test (iPITT) (*n* = 5–6). (**D**) The AUC value of blood glucose in iPITT (*n* = 5–6). Data were expressed as mean ± SEM. Data were analyzed by one-way ANOVA, followed by Tukey’s multiple comparisons test. * *p* < 0.05, ** *p* < 0.01, and *** *p* < 0.001 vs. high-AGE diet-fed rats.

To evaluate the effect of EBE and a high-AGE diet on glucose homeostasis, we performed OGTT and IPITT. Compared with the control group, rats in the AGEs group exhibited increases in blood glucose levels and corresponding AUC values following glucose administration and insulin injection (*p* < 0.05, Figure 2A–D), indicating impaired glucose tolerance and insulin sensitivity. Meanwhile, compared with the AGEs group, blood glucose levels and corresponding AUC values decreased in the EBE group after glucose administration and insulin injection (*p* < 0.05, Figure 2A–D). These findings demonstrated that EBE intervention mitigated the impairment of glucose tolerance and insulin sensitivity caused by a high-AGE diet.

### 3.3. EBE Ameliorates NASH in High-AGE Diet-Fed Rats

**Figure 3:**
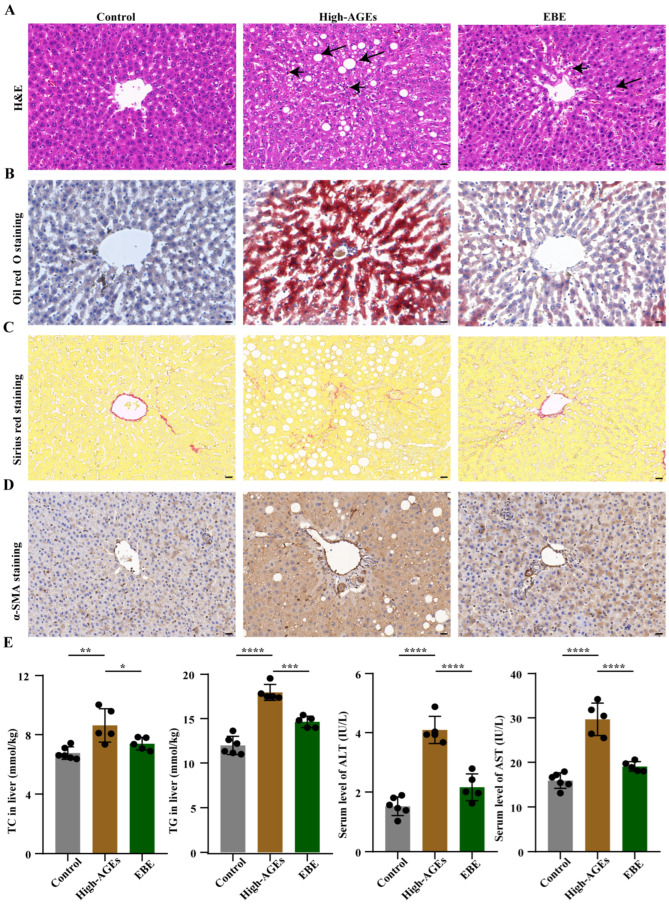
EBE intervention mitigates NASH in high-AGE diet-fed rats. (**A**) Hematoxylin and eosin (H&E) staining of liver tissue in rats (*n* = 3), with the scale bar = 20 μm. The short arrows represented the infiltration of the inflammatory factor, and the long arrows represented the lipid droplet. (**B**) Oil red O staining of liver tissue in rats (*n* = 3), with the scale bar = 20 μm. (**C**) Sirius red staining of liver tissue in rats (*n* = 3), with the scale bar = 20 μm. (**D**) Immunohistochemical staining of α-SMA in the liver tissue of rats (*n* = 3), with the scale bar = 20 μm. (**E**) The levels of total cholesterol (TC) and triglyceride (TG) in the liver and the levels of alanine aminotransferase (ALT) and aspartate transaminase (AST) in the serum (*n* = 5–6). Data were expressed as mean ± SEM. Data were analyzed by one-way ANOVA, followed by Tukey’s multiple comparisons test. * *p* < 0.05, ** *p* < 0.01, *** *p* < 0.001, and **** *p* < 0.0001 vs. high-AGE diet-fed rats.

To explore the effect of a high-AGE diet on the liver, we conducted liver pathological examination. Compared with the control group, evident lipid droplets and inflammatory cell infiltration were observed in the livers of high-AGE diet-fed rats (Figure 3A,B). However, EBE reduced liver inflammation and steatosis in high-AGE diet-fed rats (Figure 3A,B). According to Sirius red staining and α-SMA staining, increased α-SMA expression and fibrogenesis were induced by a high-AGE diet, whereas EBE inhibited liver fibrosis in high-AGE diet-fed rats (Figure 3C,D). Liver steatosis is directly associated with the accumulation of TC and TG in the liver. Furthermore, liver inflammation and fibrosis are reflected by elevations of ALT and AST. Corresponding with the pathological results, the levels of TC and TG in the liver and the levels of ALT and AST in serum increased in the high-AGE diet group when compared with the control group (*p* < 0.01, Figure 3E). However, high-AGE diet-induced increases in the levels of TC and TG in the liver and the levels of ALT and AST in serum were reduced by EBE (*p* < 0.05, Figure 3E). Overall, EBE alleviated high-AGE diet-induced liver inflammation, steatosis, fibrosis, and dysfunction in rats.

### 3.4. EBE Regulates Gut Dysbiosis in High-AGE Diet-Fed Rats

**Figure 4:**
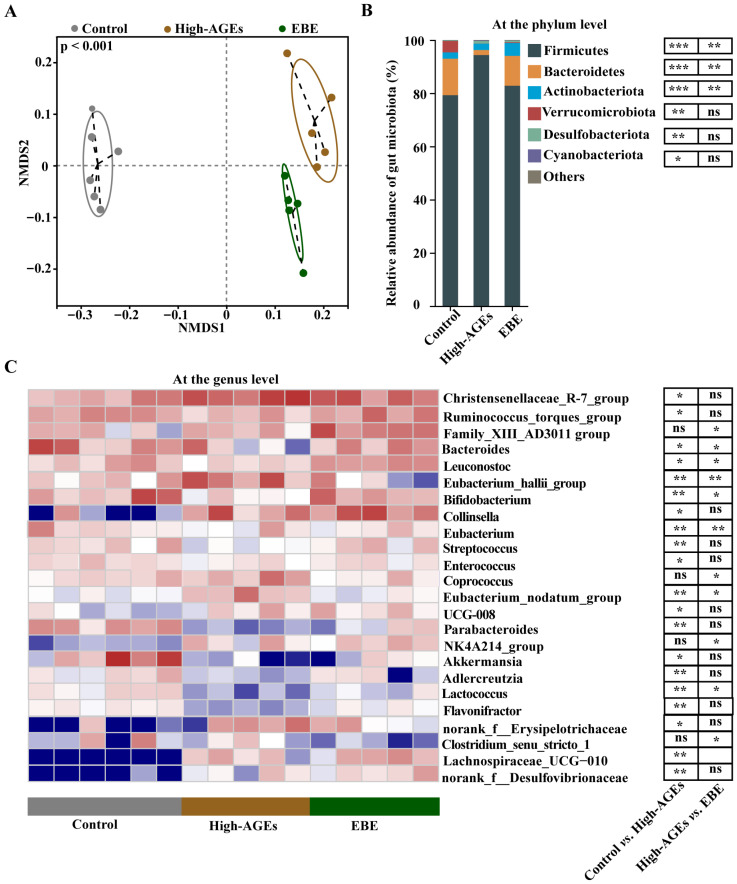
EBE intervention modulates gut dysbiosis in high-AGE diet-fed rats. (**A**) The β-diversity of the gut microbiota based on non-metric multidimensional scaling (NMDS) analysis (*n* = 5–6). (**B**) The relative abundance of gut microbiota at the phylum level (*n* = 5–6). (**C**) The relative abundance of gut microbiota at the genus level (*n* = 5–6). Data were analyzed by the Kruskal–Wallis test, followed by Dunn’s post hoc test. * *p* < 0.05, ** *p* < 0.01, and *** *p* < 0.001 vs. high-AGE diet-fed rats. ns: not significant.

16S rRNA analysis was used to investigate the effect of EBE on the gut microbiota. NMDS analysis showed that each sample was clustered within the group, and separated from other groups, which supported that the gut microbiota was obviously different among the three groups (*p* < 0.001, Figure 4A). At the phylum level, a high-AGE diet-induced increased abundance of the Firmicutes phylum and decreased abundance of the Bacteroidetes phylum were altered by EBE (*p* < 0.01, Figure 4B). At the genus level, beneficial bacteria, such as *Bacteroides*, *Leuconostoc*, *Bifidobacterium*, *Eubacterium*, *Parabacteroides*, *Akkermansia*, and *Lactococcus*, were reduced in the high-AGE diet group compared with the control group (*p* < 0.05, Figure 4C). Meanwhile, deleterious bacteria, such as *Eubacterium_hallii_group*, *Collinsella*, and *Eubacterium_nodatum_group* were elevated in the high-AGE diet group compared with the control group (*p* < 0.05, Figure 4C). However, the high-AGE diet-induced decreases in the abundance of *Bacteroides*, *Leuconostoc*, *Bifidobacterium*, *Eubacterium*, *Lactococcus* and increases in the abundance of *Eubacterium_hallii_group*, *Collinsella*, *Coprococcus*, and *Eubacterium_nodatum_group* were ameliorated by EBE (*p* < 0.05, Figure 4C). Collectively, EBE intervention modulated gut dysbiosis in high-AGE diet-fed rats.

### 3.5. EBE Increases Fecal and Serum SCFA Levels in High-AGE Diet-Fed Rats

**Figure 5:**
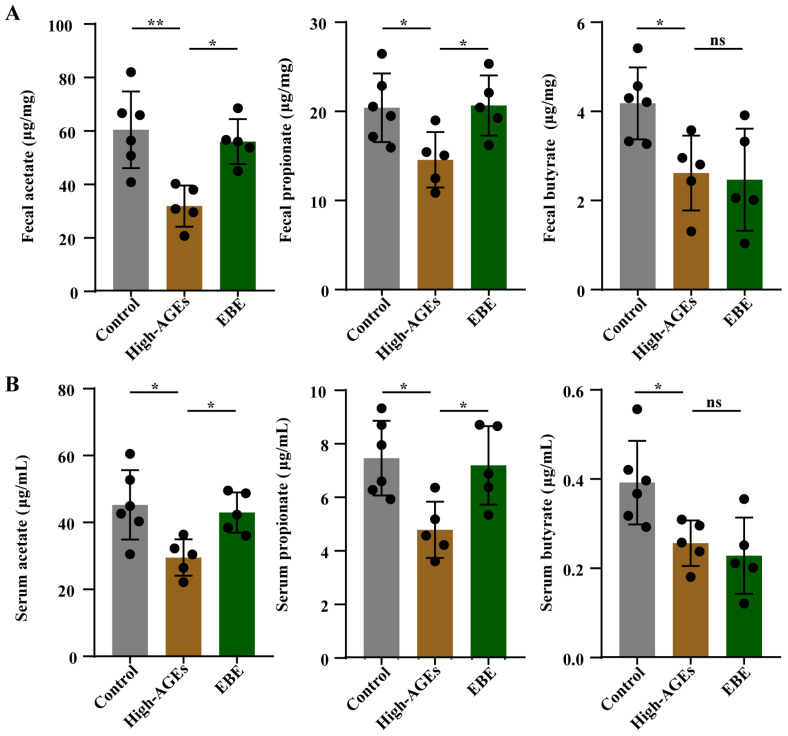
EBE intervention promotes the production of SCFA in high-AGE diet-fed rats. (**A**) The levels of acetate, propionate, and butyrate in feces (*n* = 5–6). (**B**) The levels of acetate, propionate, and butyrate in the serum (*n* = 5–6). Data were expressed as mean ± SEM. Data were analyzed by one-way ANOVA, followed by Tukey’s multiple comparisons test. * *p* < 0.05 and ** *p* < 0.01 vs. high-AGE diet-fed rats. ns: not significant.

We then explored the impacts of EBE on the common microbial metabolites—SCFA. The levels of acetate, propionate, and butyrate in feces and serum were reduced in the high-AGE diet group compared to those in the control group (*p* < 0.05, Figure 5A,B). High-AGE diet-induced decreases in acetate and propionate levels in feces and serum were alleviated by EBE (*p* < 0.05, Figure 5A,B). EBE had no effect on the level of butyrate in feces and serum (*p >* 0.05, Figure 5A,B). These results demonstrated that EBE intervention promoted the production of acetate and propionate in high-AGE diet-fed rats.

### 3.6. EBE Modulates SCFA Receptor and Suppresses the Activation of HMGB1/RAGE/NF-κB Signaling Pathway in the Liver of High-AGE Diet-Fed Rats

**Figure 6:**
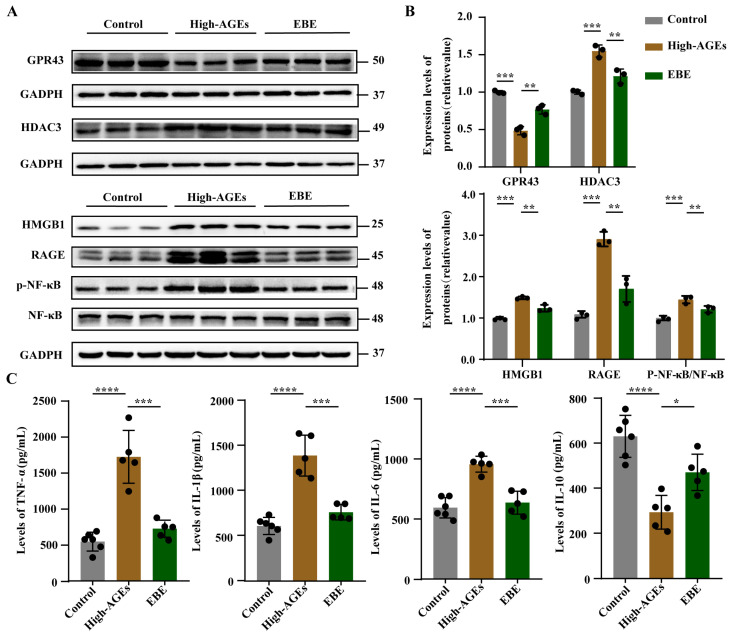
EBE modulated SCFA receptor and inhibited the activation of HMGB1/RAGE/NF-κB signaling pathway in the liver of high-AGE-fed rats. (**A**) The protein bands of GPR43, HDAC3, HMGB1, RAGE, NF-κB, and *p*-NF-κB in the liver (*n* = 3). (**B**) Quantitative analysis of proteins in the liver (*n* = 3). (**C**) The levels of TNF-α, IL-1β, IL-6, and IL-10 in the liver (*n* = 5–6). Data were expressed as mean ± SEM. Data were analyzed by one-way ANOVA, followed by Tukey’s multiple comparisons test. * *p* < 0.05, ** *p* < 0.01, *** *p* < 0.001, and **** *p* < 0.0001 vs. high-AGE diet-fed rats.

After detecting alterations in SCFA levels, we further investigated changes in SCFA receptors and their downstream signaling pathways in the liver. The expression level of GPR43 was downregulated, and the expression level of HDAC3 was upregulated in the high-AGE diet group compared with the control group (*p* < 0.001, Figure 6A,B). The high-AGE diet induced the downregulation of GPR43, and the upregulation of HDAC3 was significantly altered by EBE (*p* < 0.01, Figure 6A,B). The expression levels of HMGB1, RAGE, *p*-NF-*κB*, and NF-*κB* were elevated in the high-AGE diet group compared with the control group (*p* < 0.001, Figure 6A,B). A high-AGE diet induced the upregulation of HMGB1, RAGE, *p*-NF-*κB,* and NF-*κB*, and was also reduced by EBE (*p* < 0.01, Figure 6A,B). In addition, the levels of TNF-α, IL-1β, and IL-6 increased, while the level of IL-10 decreased in the high-AGE diet group compared with the control group (*p* < 0.0001, Figure 6C). The high-AGE diet-induced changes in inflammatory factor levels were mitigated by EBE (*p* < 0.05, Figure 6C). Overall, EBE modulated SCFA receptor and inhibited the activation of HMGB1/RAGE/NF-κB signaling pathway in the liver caused by the high-AGE diet.

## 4. Discussion

This study demonstrated that a long-term high-AGE diet caused liver inflammation, steatosis, fibrosis, and dysfunction in rats. EBE attenuated liver inflammation, steatosis, fibrosis, and dysfunction in high-AGE diet-fed rats. EBE regulated gut dysbiosis and promoted SCFA production in high-AGE diet-fed rats. EBE regulated SCFA receptor and inhibited the activation of HMGB1/RAGE/NF-κB signaling pathway in the liver of high-AGE diet-fed rats.

High-temperature-baked foods represent a primary source of exogenous AGEs [24]. Although only approximately 10% of dietary AGEs are absorbed, they are widely detected in the circulation and various tissues, including skin, kidney, and liver [4,25]. In line with these previous studies, we also found that a high-AGE diet increased the levels of AGEs in the serum and liver. Intriguingly, though a high-AGE diet increased free-/bound-CML levels, it exhibited no effect on free-/bound-CEL levels in the liver. It might be because CML had a stronger affinity for the liver than CEL [26]. The accumulation of AGEs might have adverse effects on their target tissues. A previous study found that 39 weeks of high-AGE diet intake (TestDiet 58G7, baked at 120 °C for 15 min) contributed to liver steatosis, but did not cause liver inflammation in mice [27]. At present, we observed that 80 weeks of high-AGE diet intake (AIN-93M, baked at 150 °C for 30 min) triggered liver steatosis and inflammation in rats. This discrepancy might be related to differences in the duration of high-AGE diet intake and the types and baking times of the diets. In addition, the positive expression level of α-SMA staining and Sirius red staining supported the development of liver fibrosis in rats fed with a long-term high-AGE diet. These results supported that the long-term high-AGE diet caused NASH in rats.

Except for a small part of AGEs that are directly absorbed into the circulation, the majority of AGEs are transferred into the colon and interact with the gut microbiota [11]. According to our previous study, AGEs accumulated in the colon and duodenum after high-AGE diet feeding [22]. Emerging studies have shown that dietary AGEs induce the alteration of gut microbiota and exert a vital function in disease pathogenesis [10,28]. Consistent with prior research [12,29], we also observed that dietary AGEs decreased the abundance of beneficial bacteria, such as *Bacteroides*, *Bifidobacterium*, *Akkermansia*, and *Lactococcus*, which exerted anti-inflammatory effects and mitigated liver steatosis, inflammation, and fibrosis in NASH mice induced by a high-fat diet [30]. Meanwhile, we noted that dietary AGEs enriched the abundance of deleterious bacteria, such as *Collinsella* and *Eubacterium_nodatum_group*, which has been proven to be positively related to pro-inflammatory response and linked to the progression of liver steatosis, inflammation, and fibrosis [31,32]. In addition, dietary AGEs reduced the levels of microbial metabolites-SCFA, which had an antagonistic effect on alleviating liver steatosis and inflammation in mice administered a methionine- and choline-deficient diet [33]. These results indicated that dietary AGEs-induced gut dysbiosis was involved in the development of NASH.

Anthocyanins are one of the common phytochemicals that inhibit AGEs formation and accumulation [34]. In this study, we found that EBE, an anthocyanin-rich extract, reduced AGEs accumulation in the circulation and liver. Moreover, EBE alleviated liver inflammation, steatosis, fibrosis, and dysfunction caused by a high-AGEs diet. Owing to the low bioavailability of anthocyanins and their positive effects on regulating gut microbiota [35], we preferred to explore the possible effect and mechanism of EBE in a high-AGEs diet-induced NASH through the gut microbiota. As expected, a high-AGEs diet-induced increase in Firmicutes and decrease in Bacteroidetes, which were associated with the development of NAFLD [36], were mitigated by EBE. In addition, we found that EBE increased the abundance of beneficial bacteria, such as *Bifidobacterium*, *Eubacterium*, *Lactococcus*, and reduced the abundance of deleterious bacteria, such as *Eubacterium_hallii_group*, *Collinsella*, and *Eubacterium_nodatum_group* in high-AGEs diet-fed rats. We proposed that anthocyanins, the key active components in EBE, function as prebiotic-like substances by reaching the colon intact and selectively promoting the growth of beneficial bacteria. This notion was supported by our findings and consistent with previous studies showing that anthocyanins from other botanical sources (e.g., blueberries and purple–red rice) served as favorable carbon sources for taxa like Bifidobacterium and Lactococcus [19,20,37]. Furthermore, a recent comparative study on polyphenol-rich extracts also confirmed that flavonoid components, particularly anthocyanins, played a pivotal role in balancing gut microbiota [35]. Emerging studies have indicated that *Bifidobacterium*, *Eubacterium*, and *Lactococcus* promoted the production of SCFA, and *Eubacterium_hallii_group*, *Collinsella*, *Coprococcus*, and *Eubacterium_nodatum_group* reduced the production of SCFA [38,39]. Thus, we detected the levels of SCFA and found that the levels of acetate and propionate increased in the feces and serum due to EBE intervention. Previous studies have found that SCFA exerted anti-inflammatory effects through interacting with their specific receptors, such as GPR43 and GPR109a [40,41]. In line with these studies, we observed that the expression of GPR43 was upregulated in the liver due to EBE intervention. Given that there was no change in the level of butyrate, we did not detect the level of GPR109a, which was a specific receptor for butyrate [42]. The activation of GPR43 and its downstream HDAC3 might downregulate the activation of HMGB1/RAGE/NF-κB signaling pathway, finally inhibiting pro-inflammatory responses [43]. Indeed, we found that the levels of TNF-α, IL-1β, and IL-6 decreased, and the level of IL-10 increased in the liver after EBE intervention. These results demonstrated that EBE promoted the levels of SCFA, which might suppress the activation of downstream HDAC3 and HMGB1/RAGE/NF-κB signaling pathway through their receptors GPR43, thereby reducing pro-inflammatory responses and ultimately alleviating the development of NASH caused by a high-AGEs diet.

In the present study, we have uncovered the impact of the longest-duration exposure to a high-AGEs diet on liver health to date. In addition, we have preliminarily proved that EBE had a protective effect on high-AGEs diet-induced NASH through SCFA and its regulatory signaling pathway. This study had several limitations. Although a high-temperature-baked diet mimicked human intake of AGEs-abundant foods, we cannot exclude the possibility that high-temperature-induced depletion of heat-labile nutrients (especially vitamins) and generation of other compounds (especially acrylamide) influence our experimental results. Fecal microbiota transplantation experiments and SCFA interventions are required for investigating the crucial role of gut microbiota and its metabolites in the protective effect of EBE on NASH caused by a high-AGEs diet. More detailed mechanisms of SCFA in NASH caused by a high-AGEs diet can be further identified through transcriptomics or proteomics. While animal studies have shown that EBE exerts beneficial effects on NASH, it is crucial to note the limitations posed by species differences. Consequently, extensive clinical trials will be necessary to establish a safe and effective dosage for human patients.

## 5. Conclusions

Long-term high-AGE diet caused NASH in rats. EBE enhanced the abundance of SCFA-producing bacteria and promoted SCFA production. These SCFAs might combine with the GPR43 receptor and further inhibit the activation of the downstream HDAC3 and HMGB1/RAGE/NF-κB pathway, thereby alleviating NASH induced by a high AGE diet in rats. 

## Data Availability

The original data presented in the study are included in the article/Supplementary Materials: Further inquiries can be directed to the corresponding author.

## Supplementary Materials

The following supporting information can be downloaded at: https://www.mdpi.com/article/10.3390/nu17243918/s1, Figure S1. Effects of EBE intervention on the body weights and food intake of high-AGEs diet-fed rats. Figure S2. Raw images of Western blotting analysis.
