# Supplementary material for: European Bilberry Extract Ameliorates Dietary Advanced Glycation End Products-Induced Non-Alcoholic Steatohepatitis in Rats via Gut Microbiota and Its Metabolites
Source: Nutrients. 2025 Dec 15;17(24):3918. doi: 10.3390/nu17243918 (PMC12735947; doi:10.3390/nu17243918)
Supplement: Supplementary file 1 [file nutrients-17-03918-s001.zip › Supplementary information.pdf]

Supplementary data

European bilberry extract ameliorates dietary advanced glycation end products-induced non-alcoholic steatohepatitis in rats via the gut microbiota and its metabolites

The effects of EBE on the body weights and food intake of rats fed with a high-AGEs diet

During the whole period, there were no significant differences in body weights and food intake between the control group and the high-AGEs diet group (*P* > 0.05, Fig.1A-B). Similar trends in body weights and food intake were observed between the high-AGEs group and the EBE group (*P* > 0.05, Fig.1A-B). These results illustrated that long-term EBE intervention and high-AGEs diet had no impact on the body weights and food intake of rats.

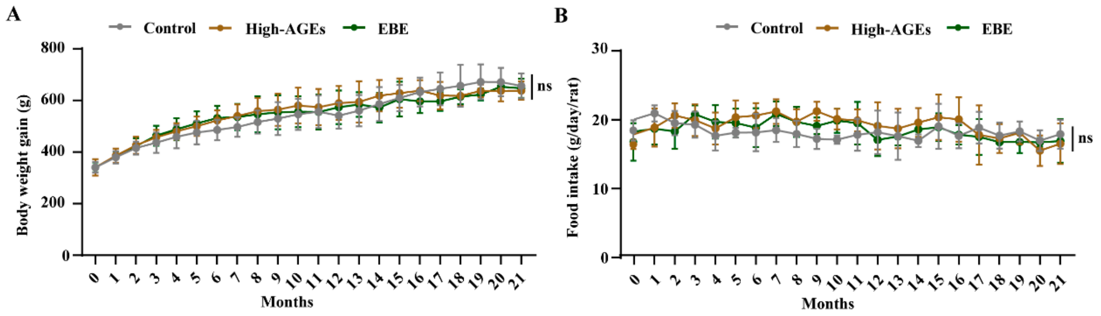

Figure S1. Effects of EBE intervention on the body weights and food intake of high-AGEs diet-fed rats. (A) Body weights (n=5-6). (B) Food intake (n=5-6). Data were expressed as mean ± SEM. Data were analyzed by one-way ANOVA, followed by Tukey's multiple comparisons test. ns: not significant.

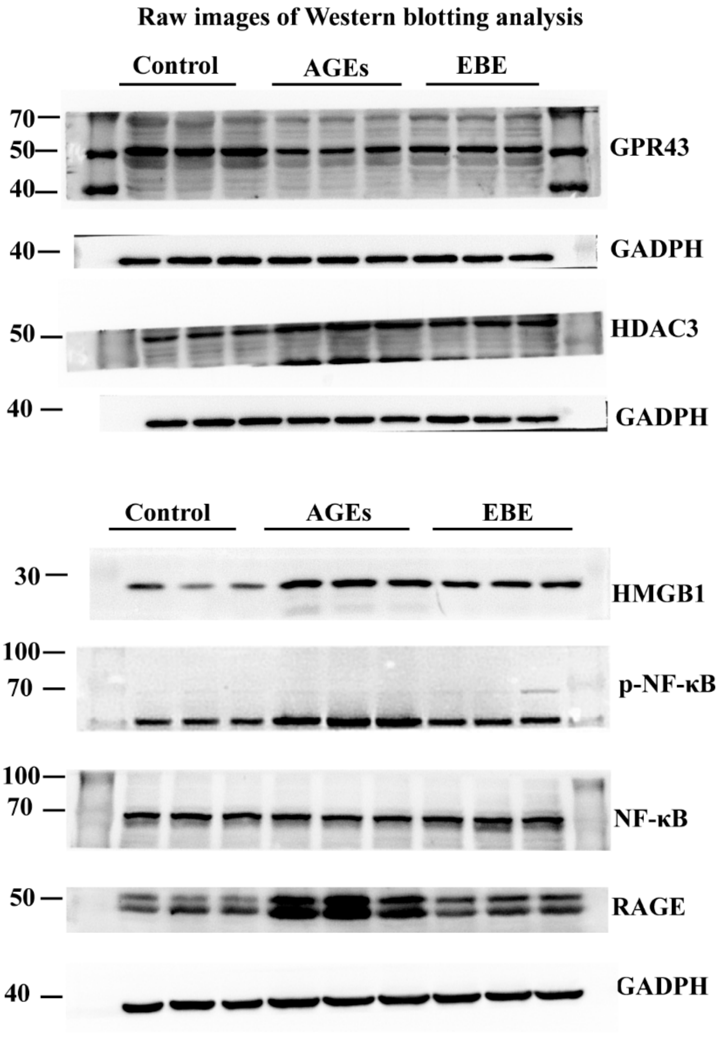

Figure S2. Raw images of Western blotting analysis.
