# Supplementary figures and images for: European Bilberry Extract Ameliorates Dietary Advanced Glycation End Products-Induced Non-Alcoholic Steatohepatitis in Rats via Gut Microbiota and Its Metabolites
Source: Nutrients. 2025 Dec 15;17(24):3918. doi: 10.3390/nu17243918 (PMC12735947; doi:10.3390/nu17243918)

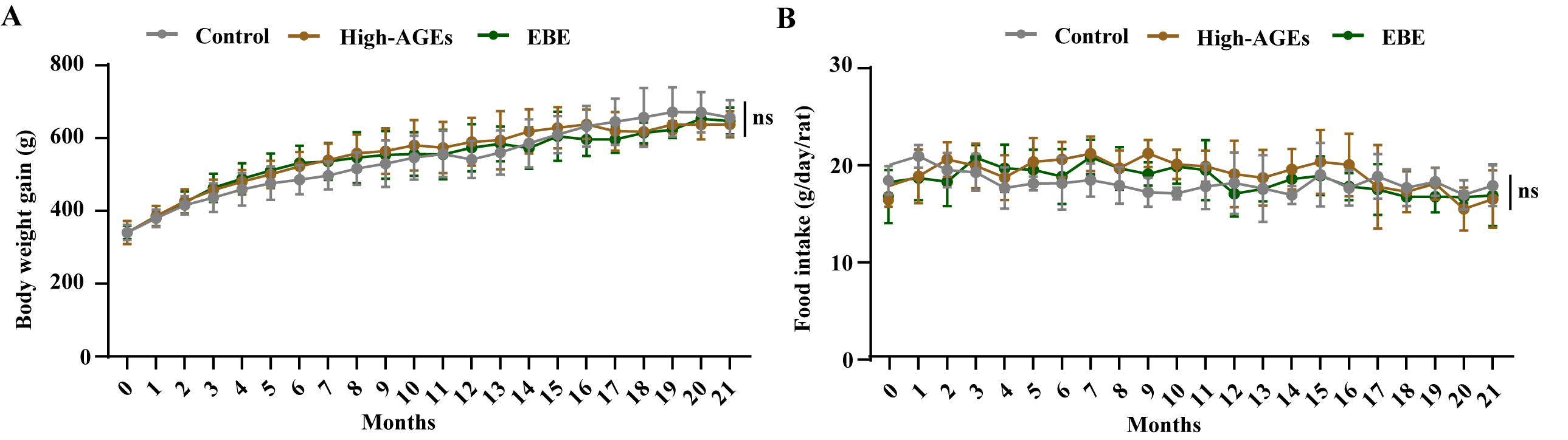

Supplement: Supplementary file 1 [file nutrients-17-03918-s001.zip › Figure S1.tif]

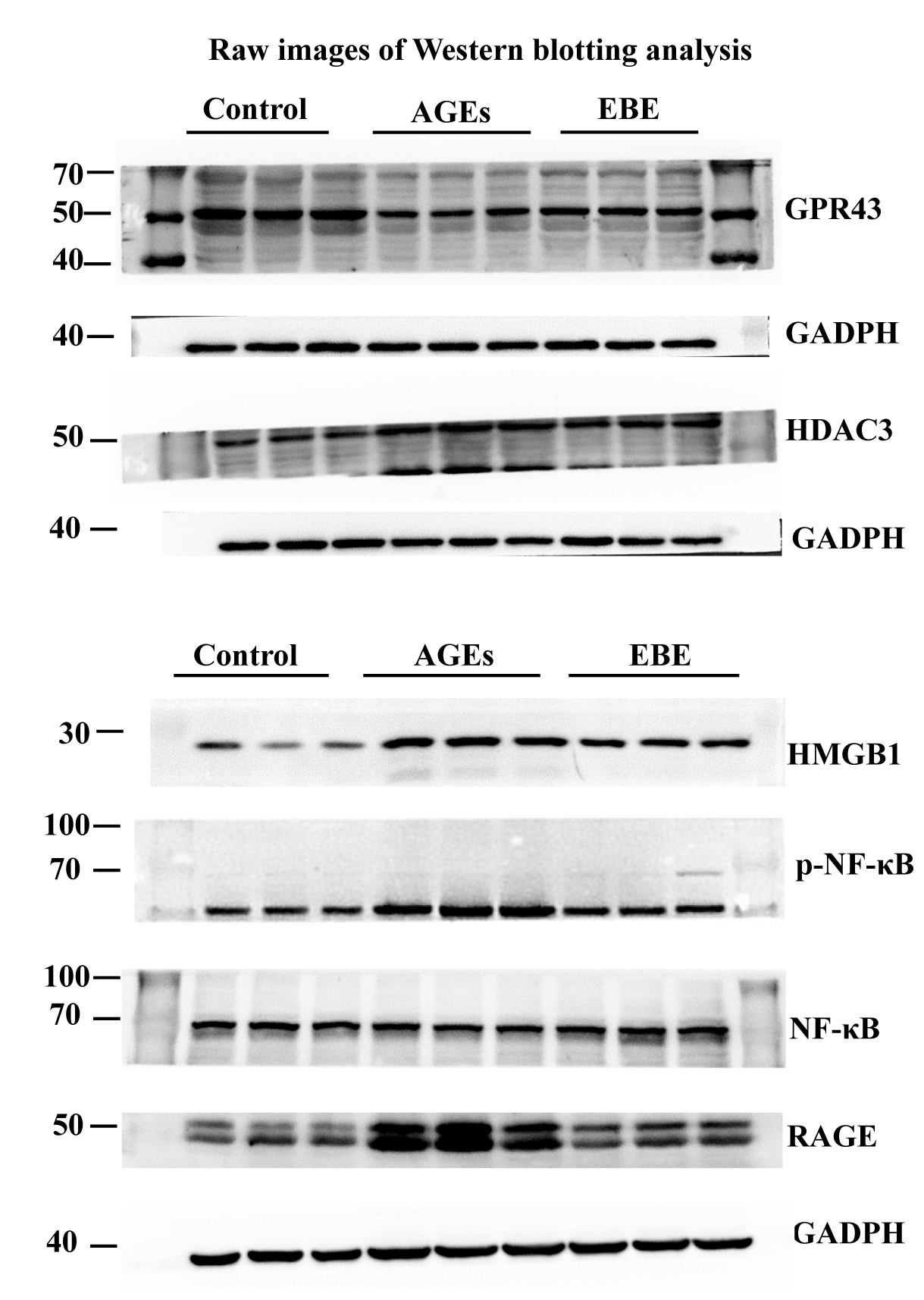

Supplement: Supplementary file 1 [file nutrients-17-03918-s001.zip › Figure S2.tif]
